# Binding mechanism of full-length Aβ40 peptide to a mixed lipid bilayer

**DOI:** 10.3389/fchem.2024.1367793

**Published:** 2024-02-21

**Authors:** Ke Wang, Wensheng Cai

**Affiliations:** Research Center for Analytical Sciences, College of Chemistry, Tianjin Key Laboratory of Biosensing and Molecular Recognition, Nankai University, Tianjin, China

**Keywords:** Aβ40 peptide, lipid bilayer, binding mechanism, binding models, peptide-bilayer interactions

## Abstract

The destructive effect of Aβ peptides on membranes is an important source of its cytotoxicity in the pathogenesis of Alzheimer’s disease. We have investigated the binding mechanism between the Aβ42 peptide and bilayer in our former work. However, as another abundant form of Aβ peptides in the physiological environment, the binding mechanism between Aβ40 peptide and the lipid bilayer still remains ambiguous. Hence, we performed all-atom simulations on the Aβ40 peptides with the lipid bilayer herein using replica exchange with the solute tempering 2 method. We obtained four major binding models with the hydrophobic C-terminus as the most preferable binding region. Hydrophobic residues and positively charged residues are the principal residues involved in the peptide-bilayer interactions. Aβ40 peptides in our simulation mainly adopt a β-rich conformation in both bound and unbound states. Besides, we determined peptide-water interactions and found that bound peptides prefer forming hydrogen bonds with water molecules than unbound peptides. Our findings herein may provide new insights for the in-depth understanding of the membrane-destructive mechanism of Aβ peptides.

## 1 Introduction

The formation of senile plaques composed of Aβ peptides is one of the main hallmarks of Alzheimer’s disease (AD) (Guillozet et al., 2003; Chabrier et al., 2012; Bloom, 2014). According to the mainstream view of the “amyloid cascade hypothesis,” the excessive accumulation and abnormal aggregation of Aβ peptides are recognized as the key factors in the onset and progression of AD (Hardy and Selkoe, 2002; Shankar et al., 2007; Sondag et al., 2009; Ferreira and Klein, 2011; Barage and Sonawane, 2015; Awasthi et al., 2016; Minter et al., 2016). Aβ is an intrinsically disordered peptide of 36–43 residues cleaved from amyloid precursor protein, with Aβ40 composed of 40 residues and Aβ42 composed of 42 residues as two predominant forms in physiological conditions (Murphy and LeVine, 2010). Aβ peptides and their products of low aggregation level, such as dimers and trimers, are toxic to the neuron and can cause neuroinflammation and further induce synaptic plasticity impairment and synapse loss (Shankar et al., 2007; Selkoe, 2008; Ferreira and Klein, 2011; Bartolotti et al., 2016; Minter et al., 2016; Mueller-Schiffmann et al., 2016). Abundant evidence shows that many pathogenic features, such as tau hyperphosphorylation, acetylcholine deficiency, oxidative stress, and inflammation, are in the downstream of Aβ pathway (Sondag et al., 2009; Chabrier et al., 2012; Lesne et al., 2013; Bloom, 2014; Amar et al., 2017; Cheignon et al., 2018). Therefore, the investigation of the toxic mechanism of Aβ peptides is vital for the understanding of the pathological mechanism of AD.

Recent studies have demonstrated that the interaction between Aβ peptides and lipid membrane is an important source of its cytotoxicity to AD (Kremer and Murphy, 2003; Arispe et al., 2007; Serra-Batiste et al., 2016; Lindberg et al., 2017; Sparr and Linse, 2019; Ciudad et al., 2020). On the one hand, the membrane can accelerate the aggregation of Aβ peptides by acting as a catalytic site for Aβ nucleation (Kremer and Murphy, 2003; Bokvist et al., 2004; Bokvist and Grobner, 2007; Lindberg et al., 2017; Sparr and Linse, 2019). The aggregation rates of Aβ in a membrane environment are faster than those in bulk solution (Kremer and Murphy, 2003; Bokvist et al., 2004; Bokvist and Grobner, 2007; Banerjee et al., 2020). On the other hand, Aβ peptides can insert into the membrane, forming destructive channels allowing water or ions to flow (Williams and Serpell, 2011; Fantini et al., 2014; Serra-Batiste et al., 2016; Osterlund et al., 2019; Ciudad et al., 2020). Full-length Aβ(1–42) or truncated β-amyloid peptide Aβ(9–42) and Aβ(17–42) peptides can form channels or channel-like structures inside the lipid bilayer (Lin et al., 2001; Arispe et al., 2007; Jang et al., 2010; Serra-Batiste et al., 2016; Bode et al., 2017; Osterlund et al., 2019; Ciudad et al., 2020). The ion-channel-like structures are found to be toxic by inducing neurite degeneration or neuritic abnormality irrespective of their size and morph (Lin et al., 2001; Jang et al., 2010). The channels formed can elicit ion-channel currents, allow calcium uptake, and disrupt the homeostasis of calcium ions (Lin et al., 2001; Quist et al., 2005; Jang et al., 2010; Williams and Serpell, 2011; Fantini et al., 2014). Moreover, the direct interactions between Aβ peptide and bilayer are destructive to the membrane, causing membrane thinning and curvature (Wong et al., 2009; Williams and Serpell, 2011; Garcia-Vinuales et al., 2021). Aβ monomer forms α-helix structure in the membrane-like environment and binding induces a coil-to-helix structure change (Utsumi et al., 2009; Wong et al., 2009). Replica exchange molecular dynamics (REMD) simulations have been utilized to study the Aβ(10–40) monomer in the dimyristoylphosphatidylcholine (DMPC) bilayer environment and found that peptides bound with the bilayer favor the structure with central hydrophobic cluster inserted inside the bilayer (Lockhart et al., 2020). Replica exchange with solute tempering (REST) has also been performed to study the Aβ(25–35) peptide in the DMPC bilayer environment (Smith and Klimov, 2018; Smith et al., 2019; Khayat et al., 2020; Khayat et al., 2021). Interactions between Aβ peptide and bilayer can happen in residues at diverse regions, and both helix, coil, or β-strand structures have been found to exist in peptides at different membrane environments (Brown and Bevan, 2017; Fatafta et al., 2020; Fatafta et al., 2022). Besides, all-atom (AA) simulations and coarse-grained (CG) simulations have also been applied to study the interactions between trimeric or hexameric Aβ fibrils with different bilayers, finding that their binding affinity with bilayer increases with increasing cholesterol content (Agrawal et al., 2023). CG models can be performed in a large timescale due to the simplification of the system, whereas compared with AA models, they lose some key interactions such as hydrogen bonds, salt bridges, etc.

Two Aβ species, Aβ40 and Aβ42, are majorly found in physiological conditions (Murphy and LeVine, 2010; Gu and Guo, 2013; Qiu et al., 2015). The contents of Aβ40 are significantly higher than Aβ42 peptides, whereas Aβ42 peptides are more toxic and more ready to aggregate (Gu and Guo, 2013; Qiu et al., 2015). Previously, we determined the binding mechanism of the Aβ42 peptide with a mixed bilayer using all-atom conventional molecular dynamics (cMD) simulation (Wang et al., 2022). Herein, as a comparison, we carried out all-atom simulations for the full-length Aβ40 peptide with an identical mixed bilayer using the enhanced sampling method of replica exchange with solute tempering 2 (REST2). The sequence for the Aβ40 peptide was divided into four function regions according to the hydrophobicity and charges of residues analogous to our former work of Aβ42: the hydrophilic N-terminus of residues D1-K16 (NT), the central hydrophobic core of residues L17-A21 (CHC), the hydrophilic central loop region of residues E22-G29 (CL), and the hydrophobic C-terminus of residues A30-V40 (CT) (Man Hoang et al., 2014; Cao et al., 2017; Owen et al., 2018; Wang et al., 2022). We found four binding models for the Aβ40 peptide binding to the bilayer with the hydrophobic CT as the most preferable interacting region. We determined the structure features of the Aβ40 peptide of both bound and unbound states. Moreover, we investigated the interactions such as hydrogen bonds formed between peptides and lipids and explored the role of water molecules in peptide-bilayer binding.

## 2 Methods

### 2.1 System setup

**FIGURE 1:**
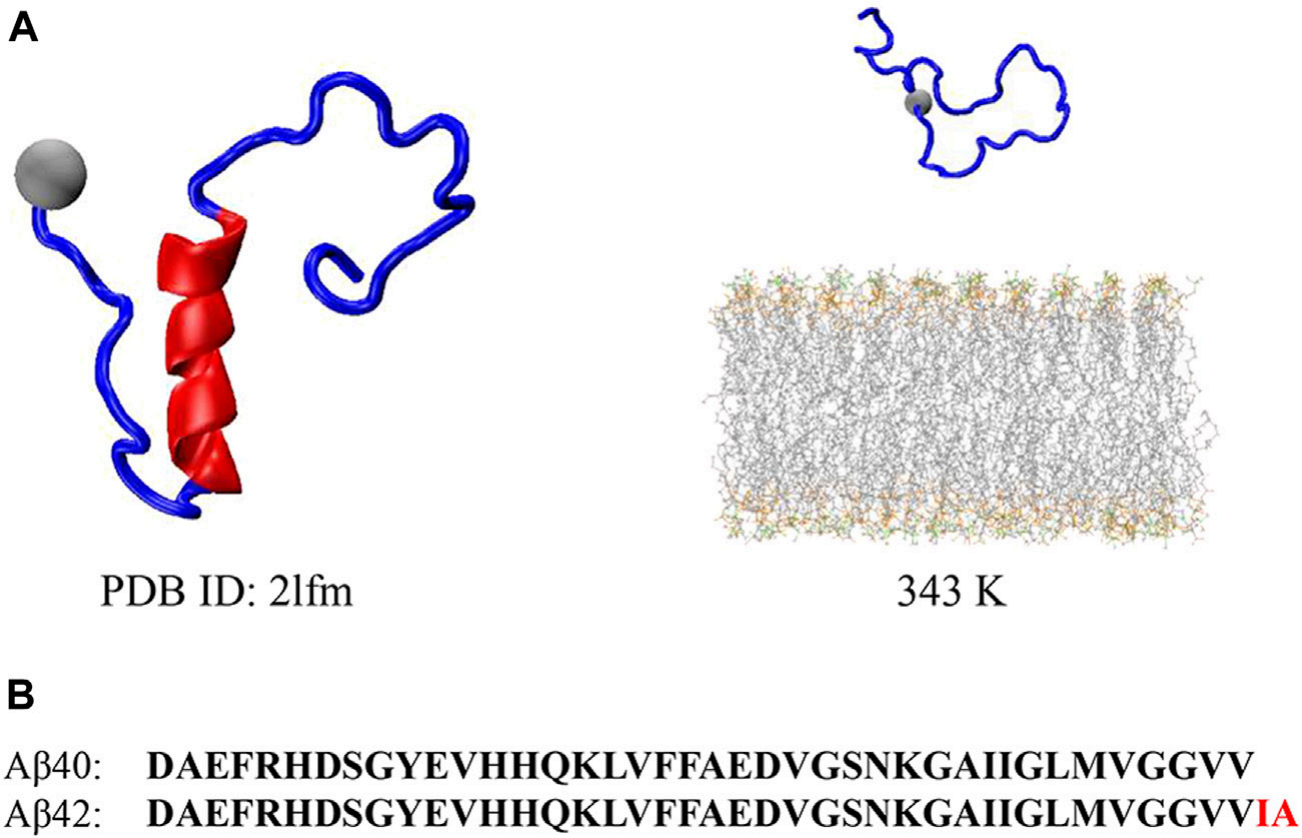
**(A)** Initial Aβ40 peptide structure obtained from Protein Data Bank for the pre-equilibrium of the peptide (left) and initial Aβ40 peptide and bilayer structure for the REST2 simulation at 343 K (right). **(B)** Sequence for the Aβ40 and Aβ42 peptides, where the two additional residues compared to Aβ40 at CT are highlighted in red in Aβ42.

Before the REST2 simulation, we performed short conventional molecular dynamic simulations for the Aβ40 peptide in solution to acquire ththe onset and progression of ADe pre-equilibrated initial conformations of the peptide for REST2. The initial structure of the full-length Aβ40 peptide is obtained from the Protein Data Bank (PDB ID: 2lfm; Figure 1A) with a helical peptide determined by nuclear magnetic resonance (Vivekanandan et al., 2011). The sequence of the Aβ40 peptide compared with the Aβ42 peptide is shown in Figure 1B. Compared to Aβ42, Aβ40 only lacks the last two hydrophobic residues I41 and A42 at the C-terminal (Tomaselli et al., 2006; Vivekanandan et al., 2011). This peptide was then under the calculation of the protonation state through the H++ web server (Gordon et al., 2005; Anandakrishnan et al., 2012) and put into a rectangle box filled with 0.15 M NaCl and TIP3P (Jorgensen et al., 1983) water molecules. The system underwent a 100 ns cMD at 343 K, and the final conformation was used as the initial peptide structure for the REST2 simulations. The peptide was placed 5.01 nm center of mass (COM) distance above the lipid bilayer and filled with 150 mM NaCl and TIP3P (Jorgensen et al., 1983) water molecules in a rectangular box. The lipid bilayer was constructed in CHARMM-GUI (Jo et al., 2009; Lee et al., 2016) with each leaflet containing 18 cholesterols (CHOL), 18 1-palmitoyl-2-oleoyl-sn-glycero-3-phospho-L-serine (POPS) lipids, and 54 1-palmitoyl-2-oleoyl-sn-glycero-3-phosphocholine (POPC) lipids, with a total of 36 CHOL, 36 POPS, and 108 POPC in the system. This POPC/POPS/CHOL bilayer in a ratio of POPC: POPS: CHOL = 3: 1: 1 is identical to our previous work (Wang et al., 2022).

### 2.2 Simulation details

All the simulations were carried out employing the GROMACS software, version 2020.6 (Van der Spoel et al., 2005), with the CHARMM36m force field (Huang et al., 2017) and a time step of 4 fs with hydrogen-mass repartitioning (Hopkins et al., 2015). The temperature coupling was dealt with using the Nosé–Hoover method while the Parrinello-Rahman barostat (Parrinello and Rahman, 1981; Nos’e and Klein, 1983) was employed for pressure coupling. The periodic boundary condition was used in all directions with the electrostatic interactions using the particle-mesh Ewald method (Essmann et al., 1995). The length of all bonds was constrained by the LINCS algorithm (Hess, 2008). The distance cutoff for the Lennard-Jones potentials and electrostatic interactions were all 1.2 nm. We also performed a 5000-step energy minimization with the steepest descent algorithm followed by 1 ns NVT and 1 ns NPT equilibration before final production runs.

The REST2 method coupled with PLUMED version 2.7.5 (Tribello et al., 2014) was used herein to explore the binding mechanism between full-length Aβ40 peptide and lipid bilayer, which provided an enhanced sampling method analogous to REMD, whereas fewer replicas were needed to achieve equal performance (Wang et al., 2011). The following expression was utilized to determine the temperature distribution in our REST2 (Jo and Jiang, 2015).

$$T_{i}=T_{\min}\exp\;\left[\frac{i\ln\left(T_{\max}/T_{\min}\right)}{N_{rep}-\;1}\right]$$ (1a)

Peptides and ions were dealt with as the “hot” region while water molecules and membranes remained cold. Exchanges between the adjacent replicas were attempted every 2 ps. Eighteen replicas were used with effective temperatures ranging between 343 K and 500 K. Each replica runs for 500 ns, resulting in a total of 9 μs simulation times for this entire work.

### 2.3 Analysis

Trajectories were analyzed using in-house codes and the GROMACS built-in programs. A free energy landscape (FEL) describing the binding process was constructed along the number of contacts and peptide-bilayer distance. Contacts between the Aβ40 peptide and lipid bilayer were determined between any heavy atoms of the peptide and bilayer within 0.5 nm. Peptide-bilayer distance was defined as the perpendicular COM distance between the peptide and the bilayer. For a closer look, the FEL describing each basin in the binding process was also constructed along the root mean square deviation (RMSD) of the peptide with respect to its initial conformation and β-sheet content. Distances between residues and membrane surfaces were generated by computing the perpendicular COM distance between each residue and bilayer and further subtracting half of the membrane thickness (2 nm). Hydrogen bond (H-bond) was defined using the criteria of 0.3 nm donor-acceptor distance cutoff and 20° angle cutoff. Free energies were defined using the following expression (1).

$$G=-\text{kT}\ln\left(P\right)$$ (1b)

where G is free energy, k is the Boltzmann constant, T is the temperature, and P is the probability of the conformations appearing in one bin. Intra-peptide contacts were computed using the 0.5 nm cutoff within the heavy atoms of residues and the contact probabilities were the average of all peptides in the ensemble. For the REST2 simulation, the first 200 ns trajectory of each replica was discarded to avoid initial transients, that is, only the 200–500 ns trajectory of each replica was utilized for analysis. All the secondary structure content calculation, H-bond analysis, and snapshot generation were carried out using the VMD software (Humphrey et al., 1996).

## 3 Results and discussion

### 3.1 Binding models explored by REST2

The REST2 method was used herein to explore the binding mechanism between Aβ40 and the bilayer. The lipid bilayer is the POPC/POPS/CHOL membrane used in our previous work (Wang et al., 2022). Similarly, this membrane was used here based on its characteristic of being extensively studied and close to the authentic cell membrane, as well as its simplicity (van Meer et al., 2008; Lemkul and Bevan, 2011; Lai et al., 2017; Banerjee et al., 2021). As our previous study revealed, Aβ42 peptides showed little interaction with the bilayer, and the obtained binding models were rare at the physiological temperature of 310 K, while the high temperature of 343 K could make the membranes ready to be penetrated without modifying their basic architecture (Wang et al., 2022). The interactions raised a lot at 343 K and the analyses of Aβ42 peptide-bilayer interactions were all based on the results at 343 K. Hence, with the aim of promoting Aβ40 binding to the lipid bilayer and for the convenience of direct comparison, we performed the REST2 simulation at 343 K for the Aβ40-bilayer system herein. The temperature distribution is calculated using Eq. 1a. Exchange rates between adjacent replicas were all larger than 17% (Supplementary Figure S1). The average exchange rate was 20.80% averaged over 18 replicas. The temperature trajectory for each replica sufficiently visits all the temperatures from 343 K to 500 K (Supplementary Figure S2). All the above outcomes demonstrated the sufficient sampling of our REST2 simulations. Consistent with our expectations, the peptides showed a high tendency to form interactions with bilayers at 343 K (Supplementary Figure S3). For most residues, the contact fractions were larger than 10% and the average contact fraction was 25.29%.

**FIGURE 2:**
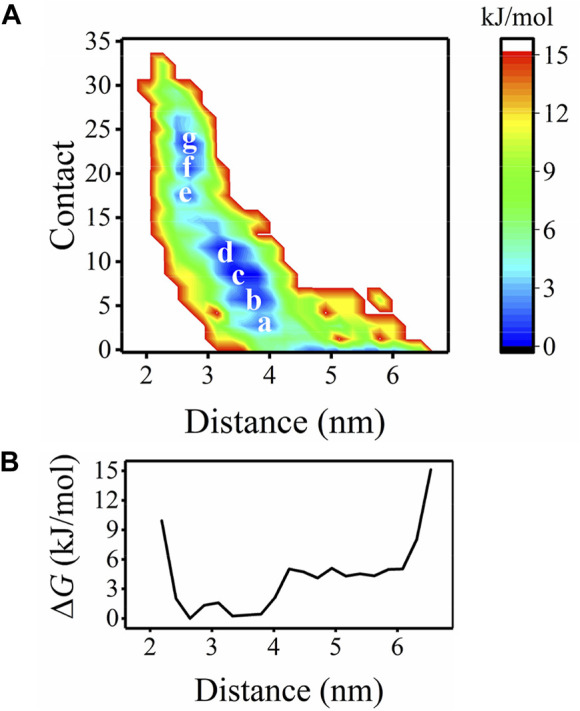
**(A)** FEL as a function of the distance and contacts between the Aβ40 peptide and bilayer with the minima are marked. **(B)** FEL in **(A)** is projected into the one dimension of distance.

First, the free energy landscape describing the position and interaction of Aβ40 with respect to the bilayer is mapped (Figure 2A). The two-dimensional (2D) FEL is constructed based on the peptide-bilayer distance and the number of peptide-bilayer contacts. Not surprisingly, as the peptide approaches the bilayer, the interactions between the peptide and bilayer increase, leading to the FEL in an L-form. Seven free energy basins, a to g, are identified. Basin c corresponds to the lowest free energy in the 2D FEL, where ΔG = 0. By projecting the 2D FEL onto the peptide-bilayer distance, we obtain the one-dimensional (1D) free energy profile (FEP, Figure 2B). From the FEP, it can be found that Aβ40 is most populated in the region of 2.65 nm (e, f, and g) and 3.33–3.56 nm (a, b, c, and d) distance. These results indicate that Aβ40 energetically favors binding with the bilayer.

**FIGURE 3:**
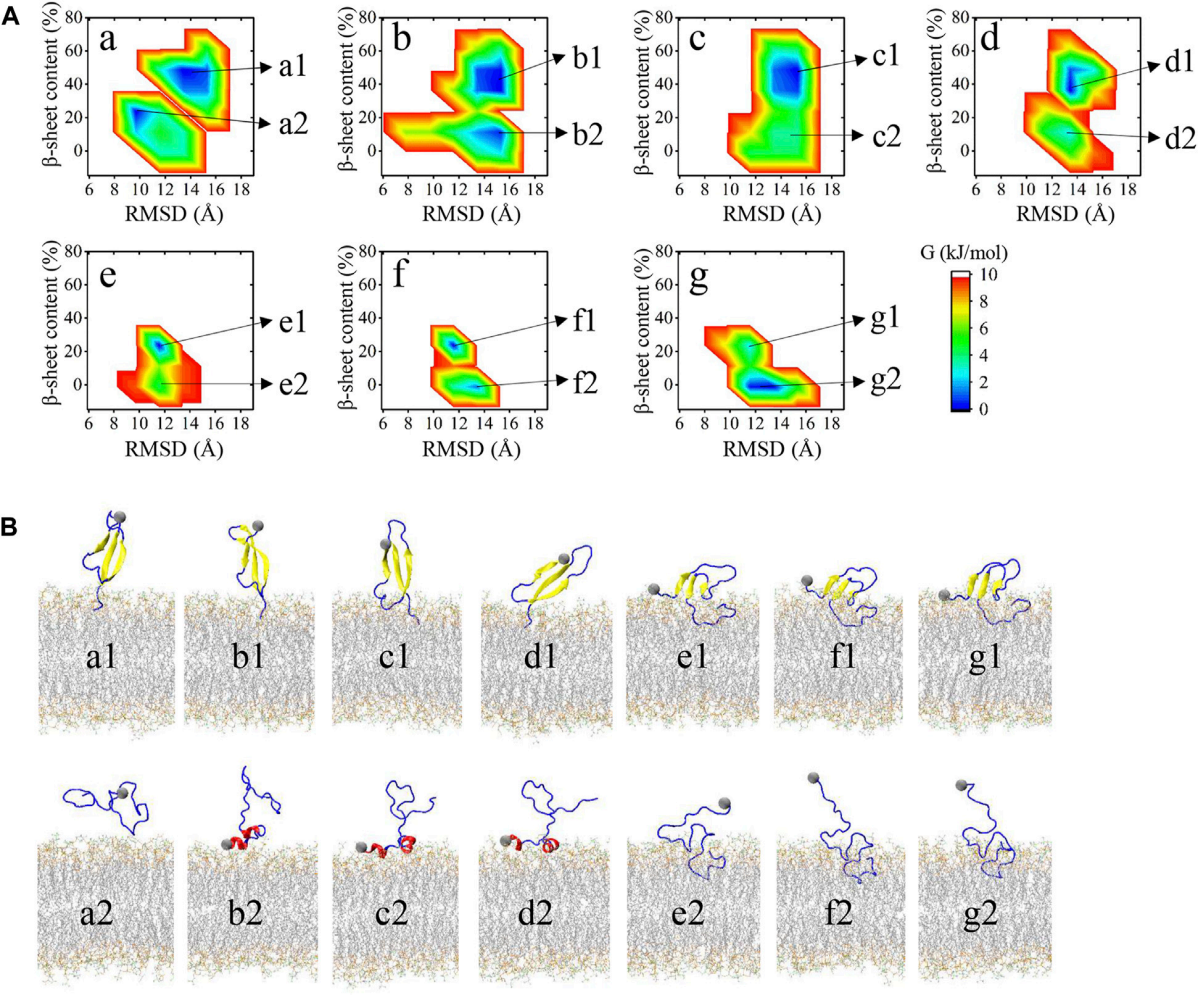
**(A)** FES for each minimum in Figure 2A as a function of RMSD between heavy atoms of the peptide with respect to its initial conformation and the β-sheet content of the peptide. Microstates in FES of each minimum are labeled at the right. **(B)** Representative structure of the microstates in **(A)**. Membrane atoms are shown in gray lines. Β-sheet structures are shown in yellow. Helix structures, including α-helix, 3–10 helix, and Pi-helix are shown in red. The remaining turn, bridge, and coil structures are shown in blue.

To further identify the bound structures distributed at the seven minima a to g characterized in Figure 2, we extracted the structural ensemble of the peptides at each minimum to construct the 2D FEL of the ensemble for each minimum to describe their structural features (Figure 3A). The FEL is mapped along the positional RMSD and β-sheet content of the peptide. As can be seen from Figure 3A, as the peptides are getting closer to the bilayer (from a to g), the β-sheet content is progressively reduced. Conformations at each minimum can be explicitly classified into two groups, and their representative structures are shown in Figure 3B. One group, labeled as a1, b1, c1, d1, e1, f1, and g1 of Figure 3B, contains bound peptides with predominantly β-sheet structure. Another group, labeled as a2, b2, c2, d2, e2, f2, and g2 of Figure 3B, contains bound peptides with predominantly helix or coil structure.

**FIGURE 4:**
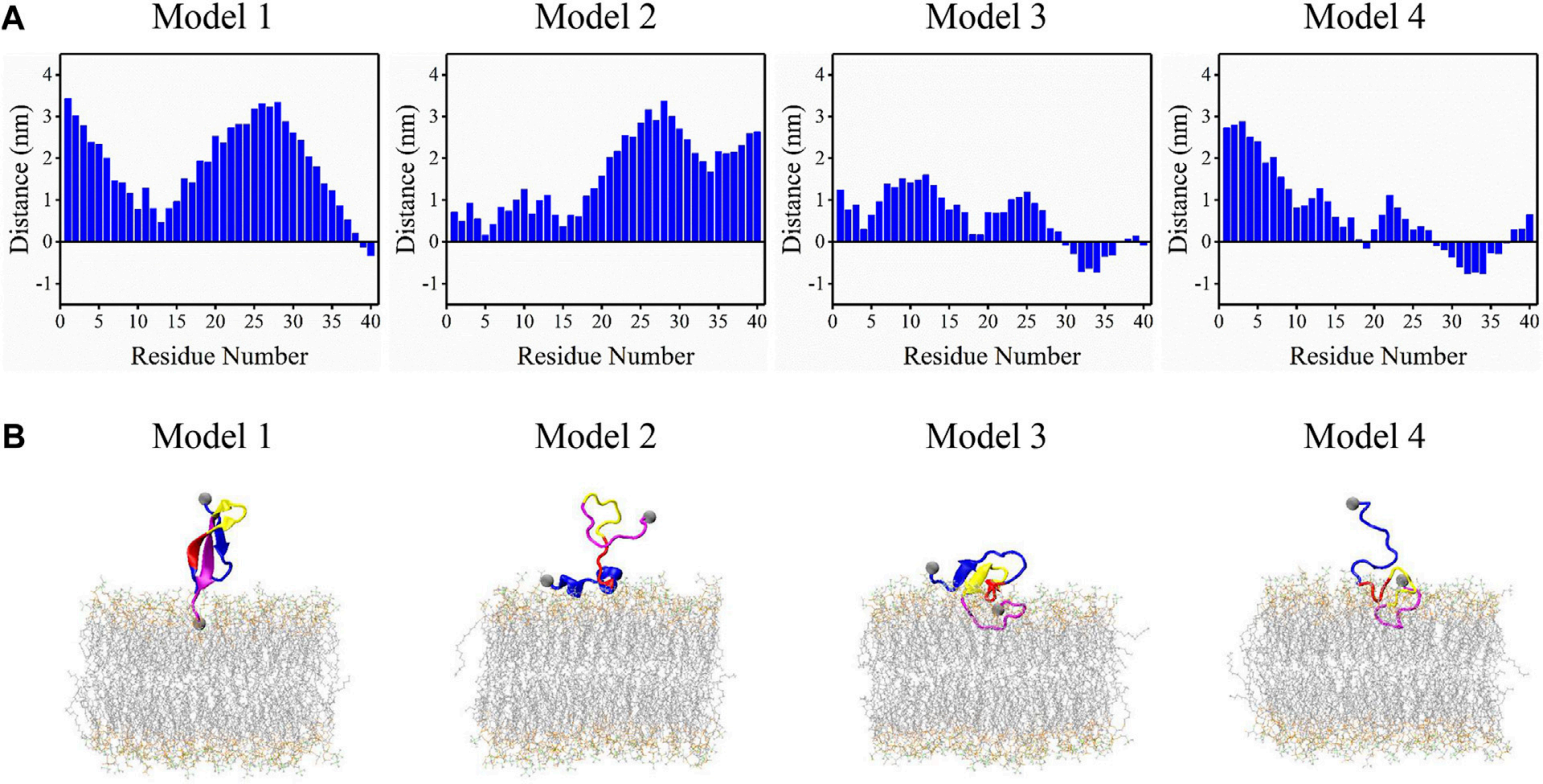
Four binding models were acquired from the REST2 simulation. **(A)** The distance between each residue and membrane surface. Negative values represent residues buried below the bilayer surface. **(B)** Corresponding structures of peptide and bilayer of each model, where the four function regions NT, CHC, CL, and CT of the peptides are colored in blue, red, yellow, and magenta, respectively.

Snapshots in Figure 3B can accurately represent the microstates in Figure 3A except for a2. a2 microstate contains peptides with few residues interacting with the membrane surface and their conformations are disordered and distinct from each other. The remaining 13 microstates can be classified into four binding models according to their structural features and membrane-interacting regions. Distances between each residue and bilayer surface of the four interacting models are shown in Figure 4A. Snapshots of peptides and bilayers are depicted in Figure 4B. Microstates a1, b1, c1, and d1 are classified into model 1, where barely several residues of CT are inserted inside the bilayer with other residues remaining in the solution. Conformations in this model adopt a β-rich structure of 30%–50% β-sheet content with three parallel β-strands as NT, CHC, and CT region each possessing one β-strand. Microstates b2, c2, and d2 are classified into model 2, where several residues of NT are lying on the membrane surface. Peptides in this model adopt two helical fragments at NT and residues at these two fragments are slightly touching the bilayer surface. Model 3 contains microstates e1, f1, and g1, where the peptides are mainly β-sheet structures with most residues of CT buried inside the bilayer, and several residues in NT and CHC are lying on the membrane surface. Compared to the β-rich structure in model 1, peptides of this model also adopt the structure with three parallel β-strands, whereas the β-sheet structures are of 10%–30% content lower than model 1 with NT, CHC, and CL region, each possessing one β-strand. Besides, residues at CT in model 3 were buried significantly deeper than in model 1. Microstates e2, f2, and g2 are classified into the most deeply buried model 4. Peptides in this model are unstructured and dominated by a random coil with the CHC and CT buried below the membrane surface while NT and CL stretch into the solution. Two to 15 residues of peptides in model 1 and model 2 contact with the bilayer in the 3.0 nm–4.0 nm peptide-bilayer distance region, whereas 16 to 25 residues in model 3 and model 4 contact and insert deeper in the 2.5 nm to the 3.0 nm distance region.

These binding models of Aβ40 were further compared with those of Aβ42 reported in our previous work (Wang et al., 2022). The results show two significant differences in their binding models. From their representative structures in Figure 4, it can be seen that Aβ40 can adopt more diverse structures of bound peptide including β-strand, helix, or unstructured structure, whereas bound peptides in Aβ42 are mostly unstructured. This difference can be attributed to the highly enhanced sampling efficiency of REST2 compared to cMD as the latter was used in the work of Aβ42. Another difference is that residues at CT frequently interact with the bilayer in Aβ40 (Models one to four in Figure 4) while showing little tendency to bind with the bilayer in Aβ42. This can be explained by the two additional residues Ile41 and Ala42 in CT of Aβ42. Due to the absence of these two residues in Aβ40, two Val residues with stronger hydrophobicity are exposed at the CT region, which can interact with the bilayer more frequently driven by the hydrophobic interactions.

**FIGURE 5:**
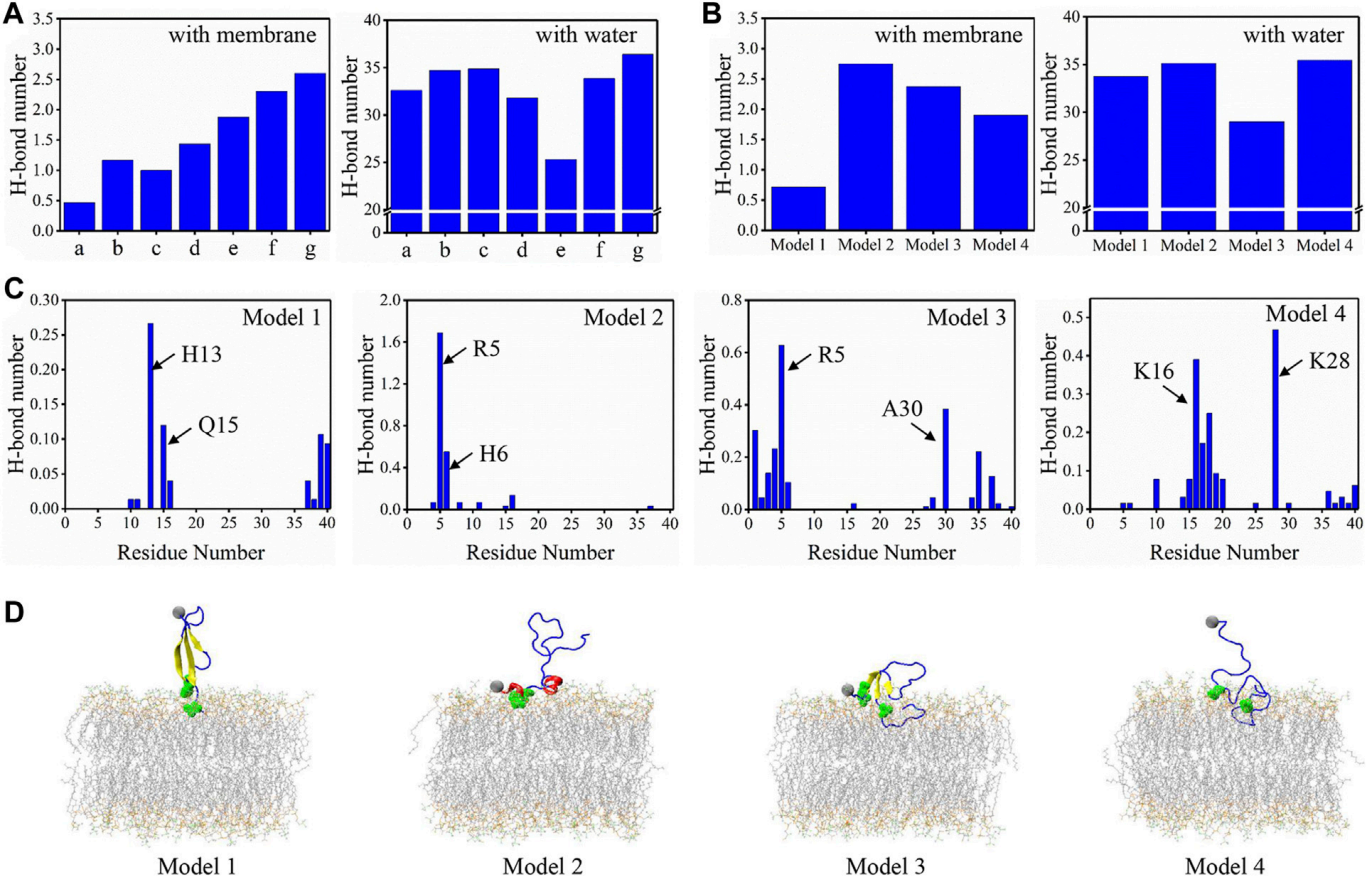
**(A)** The average number of H-bonds formed between peptide and membrane/water for minima a to g. **(B)** The average number of H-bonds formed between peptide and membrane/water for four binding models. **(C)** The average number of H-bonds formed between each residue and membrane for four binding models. The two residues forming the highest number of H-bonds are pointed in each model. **(D)** The representative conformations for four binding models with the two residues forming the highest H-bond are highlighted in green balls. The color scheme for peptide and bilayer is identical to Figure 3.

To give a deeper view of the conformation for the binding peptides, the H-bonds formed between the peptide and membrane/water for minima a to g were obtained (Figure 5A). From minima a to g, with the contacts increased, the number of H-bonds formed by Aβ40 with bilayer also shows a roughly rising trend. However, there is no obvious rising or declining trend for the H-bond formed with water. We then computed the H-bonds formed between binding models and the membrane/water (Figure 5B). The peptide-bilayer H-bonds exhibit no correlation with the peptide-water H-bonds. Conformations in model 2 form the highest number of H-bonds with the membrane but also show a high tendency to form H-bonds with water. Conformations in model 1 form the least H-bonds with the membrane. The average number of H-bonds formed by each residue with membrane was then acquired to explore the role of residues in peptide-membrane interactions in four binding models (Figure 5C). Two residues forming the highest number of H-bond with bilayer were pointed in each model. Most of these residues were charged especially for the deepest binding model of model 4, and positively charged K16 and K28 form strong H-bonds with the bilayer. Positively charged R5 residue at NT also plays a key role in forming H-bonds in models 2 and 3. We have labeled these residues intuitively in the structure of binding models in Figure 5D. They are all distributed in the peptide-bilayer interacting regions. The free energy data were computed using Eq. 1b to compare the relative stability of each microstate (Supplementary Table S1). Microstates d1, g2, and c1 showed the lowest free energy, indicating the high stability of these microstates. Free energies of other microstates were relatively high, especially for b2, c2, and d2 of model 2, suggesting the instability of this binding model.

### 3.2 Structural features of the Aβ40 peptide

**FIGURE 6:**
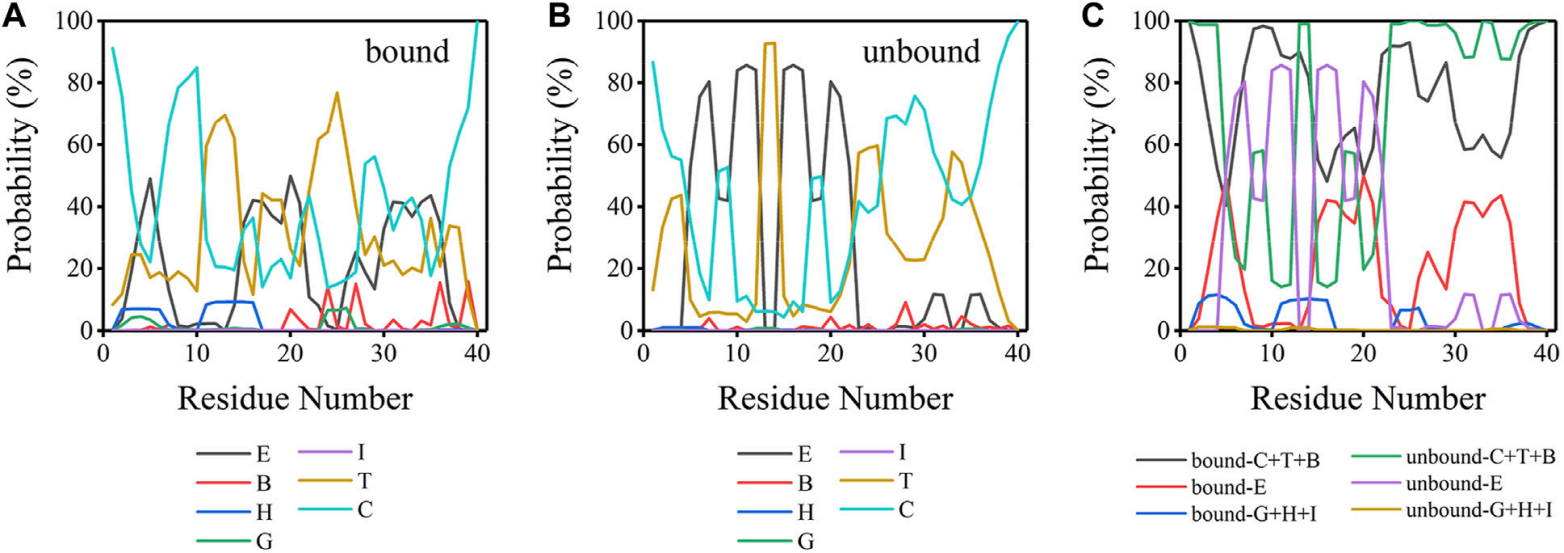
**(A)** Secondary structure probability for each residue of bound peptide. **(B)** Secondary structure probability for each residue of unbound peptide. **(C)** Secondary structure probability for each residue for the sum of coil, turn, bridge structure (C + T + B); the sum of the β-sheet (E); and the sum of 3–10 helix, α-helix, and Pi-helix structure (G + H + I) for bound and unbound Aβ40 peptides. E: extended β-sheet structure, B: bridge structure, H: α-helix structure, G: 3–10 helix structure, I: Pi-helix structure, T: turn structure, and C: coil structure.

Aβ peptides, as numerous works previously characterized, are intrinsically disordered in bulk water (Viola and Klein, 2015; Ono, 2018). The initial Aβ40 conformation used here is a partially folded structure with a helix structure spanning residues H13-D23. To explore the conformational transition of Aβ40 induced by binding to the lipid bilayer, an analysis of the secondary structure was carried out, as delineated in Figure 6. Aβ40 peptides were considered bound when their peptide-bilayer contacts in Figure 2A are larger than zero; otherwise, they are considered unbound. As can be seen from Figures 6A, B, for both the bound and unbound peptides, β-sheet (E), turn (T), and coil (C) structures were dominant. These three structures took up at least 79.50% and 90.80% of the bound and unbound peptides, respectively, and alternatively exist in each residue. In Figure 6C, the seven structures obtained were classified into three groups: the sum of the fraction for coil, turn, and bridge structure (C + T + B) was taken as the unstructured structure fraction; the fraction for β-sheet structure (E) was taken as the β-sheet structure fraction; the sum of 3–10 helix, α-helix, and Pi-helix structure (G + H + I) was taken as the helix structure fraction. Fractions of helix structure were low in both bound and unbound peptides, whereas residues E3-R5 and H13-Q15 at NT could form helix structures with fractions larger than 10% in the bound peptides. Peptides possessed more helix structure in the bound state than in the unbound state. β-sheet content (E) of both bound and unbound peptides was much higher than that of helix, especially for unbound peptides. Residues D7, E11, Q15, K16, L17, and F20 in unbound peptides adopted the β-sheet structure with fractions larger than 80%, which were all distributed at the NT and CHC regions. In the bound state, the highest fraction of β-sheet was 49.84%, which was evidently lower than the unbound state, and the residues with β-sheet structure were widely distributed in NT, CHC, CL, and CT regions. In summary, the β-sheet structure is dominant in both bound and unbound Aβ40 peptides.

### 3.3 Peptide-bilayer interactions

**FIGURE 7:**
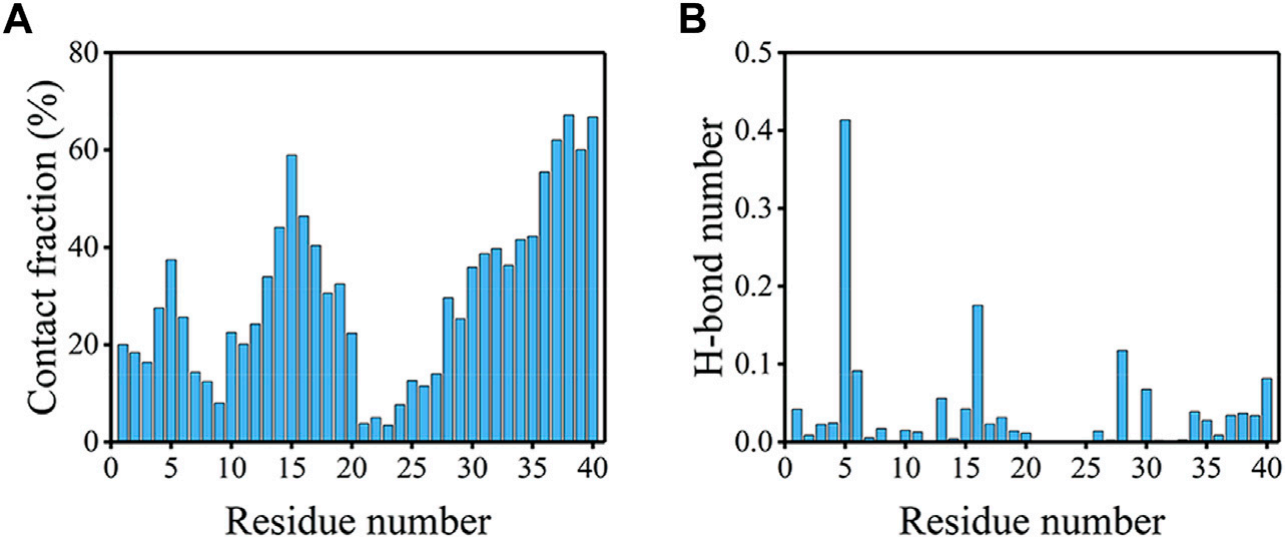
**(A)** Contact fractions of residues with the membrane in the bound peptides. When there is a contact between any of the heavy atoms of one residue and the bilayer, then this residue is considered to be in contact. **(B)** The average number of H-bonds formed between each residue and the bilayer.

To explore the driving force for the peptide binding, the peptide-bilayer contacts and H-bonds were analyzed. It is clear from Figure 7A; Supplementary Figure S4 that binding events occurred mainly in the NT, CHC, and CT regions. The contact fractions of residues in hydrophilic CL were significantly lower, especially for residues E22-N27. Residues at CT showed the highest tendency to interact with the bilayer. The top 10 residues with the highest contact fractions were G38, A40, G37, V39, Q15, V36, K16, H14, M35, and L34, which were all hydrophobic or positively charged residues. We compared the average fractions of residue-bilayer contact between Aβ40 and Aβ42 in the four sequence regions in Supplementary Figure S4 (Wang et al., 2022). It is remarkable that residues at CT in Aβ40 frequently interacted with the bilayer, whereas they showed little tendency to bind with the bilayer in Aβ42. From Figure 7B, R5 formed the highest number of H-bonds much larger than other residues, followed by K16 and K28 residues. These three residues also exhibited the same behavior in Aβ42 (Wang et al., 2022). The highest 10 types of H-bonds formed between R5, K16, and K28 residues and bilayer are provided in Supplementary Figure S5. The most populated H-bonds are formed with the side chain of POPC or POPS lipids. Like in the Aβ42-bilayer system, cholesterols also do not favor forming H-bonds with these residues (Wang et al., 2022). Overall, residues in the CT region of the Aβ40 peptide showed the highest tendency to interact with the bilayer, and H-bonds formed between positively charged residues and bilayer may drive the binding. Both hydrophobic interactions and electrostatic interactions contributed to the binding of Aβ40 to the bilayer.

### 3.4 Intra-peptide and peptide-water interactions

**FIGURE 8:**
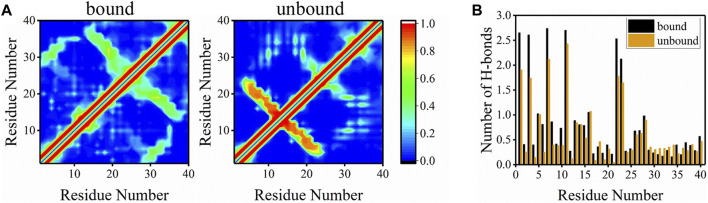
**(A)** Residue-residue contact maps of peptides in bound and unbound states. The color bar corresponding to the contact probability from 0 to 1 is shown on the right. **(B)** The average number of H-bonds formed between each residue and water molecules for bound and unbound peptides.

Besides peptide-bilayer interactions, intra-peptide and peptide-water interactions may also drive the peptide bind to the bilayer. Residue-residue contact maps for the bound and unbound Aβ40 peptides were plotted to investigate the intra-peptide interactions (Figure 8A). Unlike Aβ42, the conformations of Aβ40 in the unbound state were rather different from the bound state (Wang et al., 2022). The evident cross-diagonal formed by residues F4-V12 and residues H14-D23 in the contact map of the unbound state corresponded to the anti-parallel β-sheet structure formed at these regions. The contact probabilities in these regions were extremely high, corresponding to the high β-sheet structure of these regions in Figure 6C. A cross-diagonal represented that the β-sheet structure was also formed by residues H14-A21 and V24-V36 of the bound peptide, whereas the probabilities were not as high as the β-sheet structure in the unbound peptide. Besides, there was a band indicating the contacts formed between residues A2-H6 and residues G29-G33, which corresponded to the β-sheet interactions at these two fragments. The highest 10 contact fractions for the residue pairs (at least three residues apart) were used to characterize long-range interactions (Supplementary Figure S6).

To explore the peptide-water interactions, the average number of H-bonds formed between peptides and water molecules was counted (Figure 8B). Surprisingly, for most residues in the bound state, the average number of H-bonds formed with water was higher than residues in the unbound state except for a few residues at CT. This phenomenon occurred due to the fact that bound peptides were more exposed to water than unbound peptides and water molecules could drive the binding of peptide to the bilayer. Another reasonable explanation is that Aβ40 in extended solvent-accessible structure preferred binding with the bilayer than in the globular structure. These results suggested that water molecules played an important role in the binding of Aβ40 to membranes.

## 4 Conclusion

In the present work, we investigated the interactions between full-length Aβ40 peptide and POPC/POPS/CHOL bilayer using the REST2 method. We first explored the binding mechanism by extracting the conformations in the free energy landscape. The conformations could be classified into four binding models. Peptides in model 1 adopted a β-rich structure with only several residues of CT inserted inside the bilayer. Model 2 contained peptides with NT lying on the membrane surface and adopted two helical fragments at NT. Peptides in model 3 were also β-rich structures with most residues of CT buried inside the bilayer and several residues in NT and CHC lying on the bilayer surface. The most deeply buried model 4 contains peptides dominated by the random coil with the CHC and CT buried deeply inside the membrane. Hydrophobic CT was the region showing the highest tendency to interact with the bilayer. Residues most preferably forming H-bonds with bilayer were positively charged R5, K16, and K28 residues. Aβ40 peptides in both bound and unbound states mainly adopted the β-rich structure, whereas bound peptides showed slightly higher fractions of the helix structure than unbound peptides. We also computed the H-bonds formed between peptide and water molecules to unveil the role of water in peptide-bilayer binding. Peptides in the bound state form more H-bonds with water than in the unbound state, which showed strong proof of the vital role of water molecules in driving the peptide-membrane binding.

## Data Availability

The original contributions presented in the study are included in the article/Supplementary Material, further inquiries can be directed to the corresponding author.
