# Supplementary material for: Binding mechanism of full-length Aβ40 peptide to a mixed lipid bilayer
Source: Front Chem. 2024 Feb 21;12:1367793. doi: 10.3389/fchem.2024.1367793 (PMC10914957; doi:10.3389/fchem.2024.1367793)
Supplement: Supplementary file 1 [file DataSheet1.docx]

**Supplementary Material: Binding mechanism of full-length Aβ40 peptide to a mixed lipid bilayer**

***Ke Wang and Wensheng Cai*^*^**

Research Center for Analytical Sciences, College of Chemistry, Tianjin Key Laboratory of Biosensing and Molecular Recognition, Nankai University, Tianjin 300071, China

***Corresponding Author**: wscai@nankai.edu.cn





**Supplementary Figure S1.** Exchange rates between replica pairs for the REST2 simulations at 343 K.


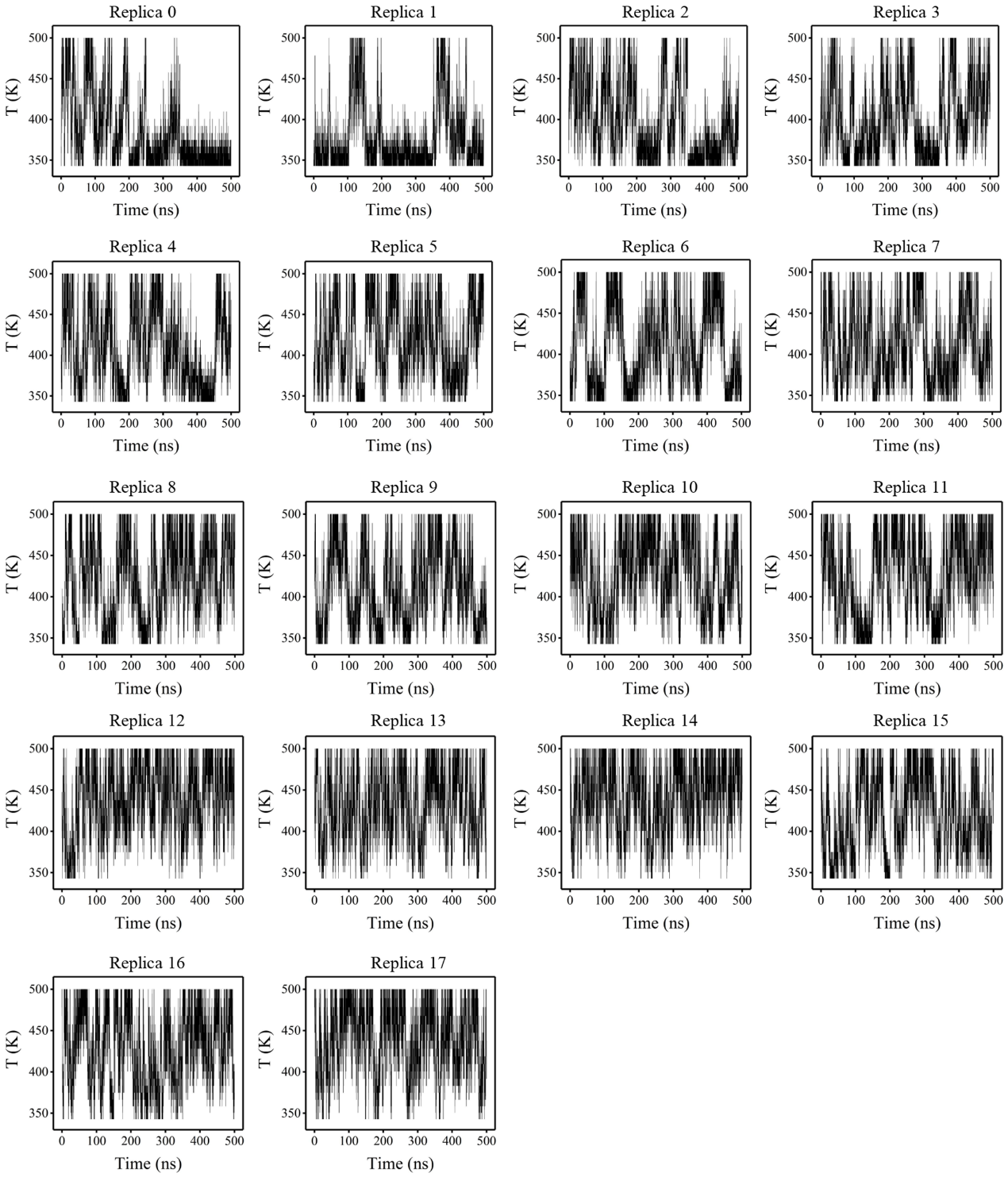


**Supplementary Figure S2.** Temperature trajectories for the 18 replicas of the REST2 simulation at 343 K. The effective temperatures range from 343 K to 500 K.


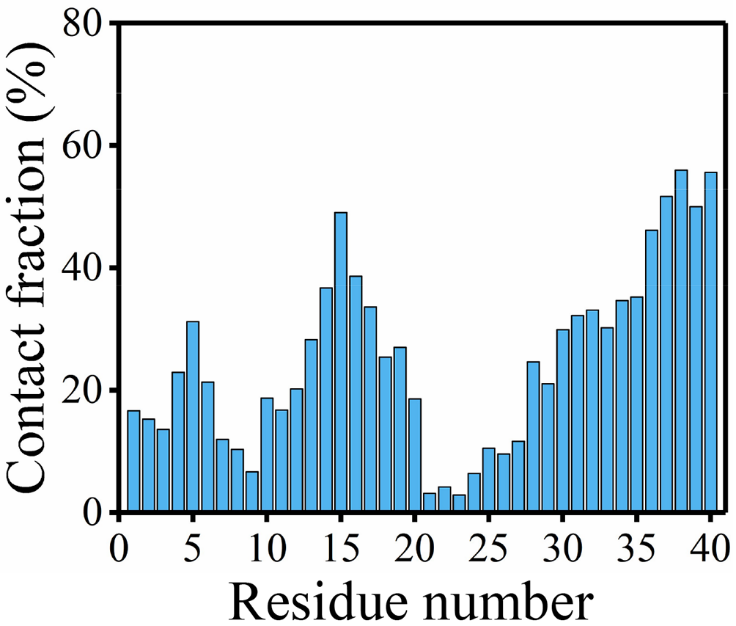


**Supplementary Figure S3.** Contact fractions of residues with the membrane. When there is a contact between any of the heavy atoms of one residue and bilayer, then this residue is considered as contacted.

**Supplementary Table S1.** Free energy (G) for microstates in Figure 3.

| Microstate | G (kJ/mol) | Microstate | G (kJ/mol) |
| --- | --- | --- | --- |
| a1 | 12.50 | a2 | 13.78 |
| b1 | 10.48 | b2 | 11.85 |
| c1 | 9.68 | c2 | 14.23 |
| d1 | 9.45 | d2 | 13.78 |
| e1 | 10.90 | e2 | 15.12 |
| f1 | 11.18 | f2 | 11.49 |
| g1 | 13.58 | g2 | 9.52 |





**Supplementary Figure S4.** Comparison of the average contact fractions of each residue with the bilayer in the four function regions for Aβ40 and Aβ42. The data of Aβ42 are adopted from our previous work (Wang et al., 2022).


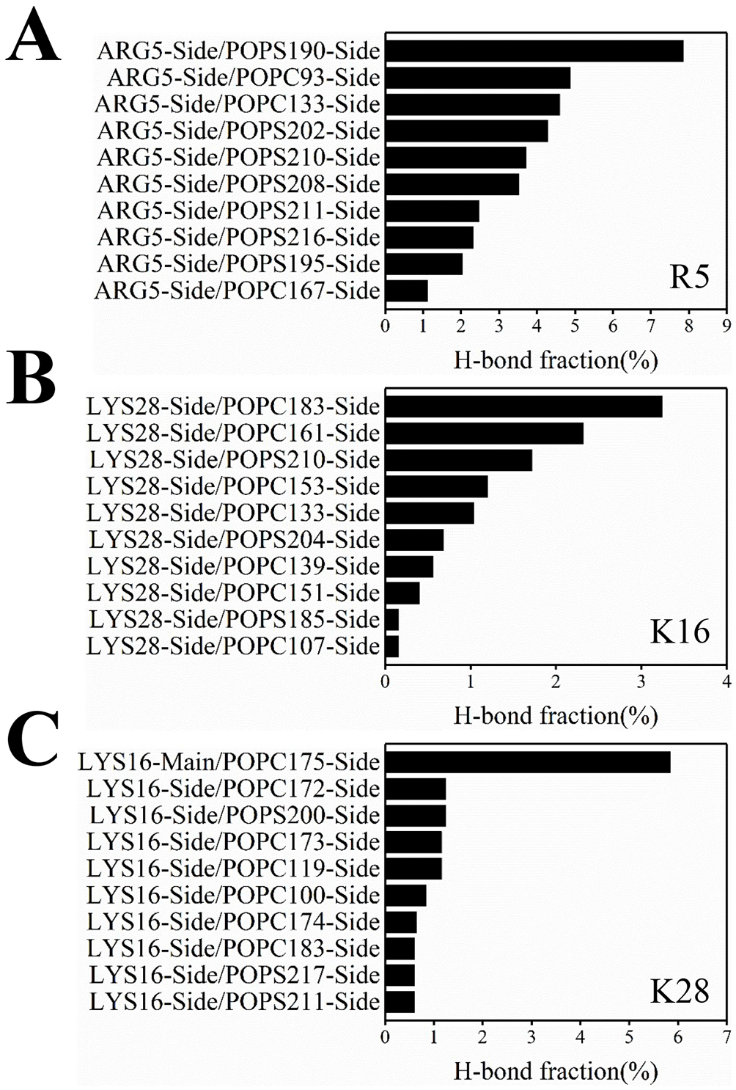


**Supplementary Figure S5.** The highest fraction of ten types of H-bonds formed between R5 (A), K16 (B), K28 (C) residues and bilayer of bound peptides.


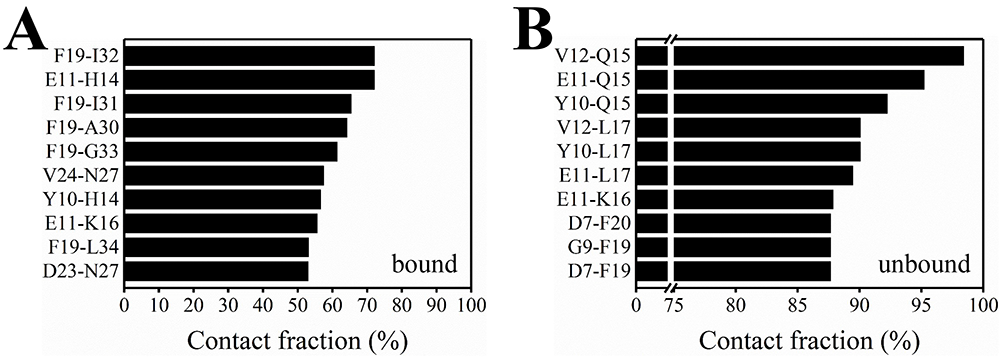


**Supplementary Figure S6.** The ten contacts with the highest proportion in bound (A) and unbound (B) peptides. The contact probabilities are computed and sorted within residue pairs that are at least three residues apart.

**References**

Wang, K., Shao, X. and Cai, W. (2022). Binding models of Aβ42 peptide with membranes explored by molecular simulations. *J. Chem Inf. Model.* 62, 6482-6493. doi:10.1021/acs.jcim.2c00444
